# Land and nature as sources of health and resilience among Indigenous youth in an urban Canadian context: a photovoice exploration

**DOI:** 10.1186/s12889-020-08647-z

**Published:** 2020-04-20

**Authors:** Andrew R. Hatala, Chinyere Njeze, Darrien Morton, Tamara Pearl, Kelley Bird-Naytowhow

**Affiliations:** 1 Department of Community Health Sciences, Max Rady College of Medicine, University of Manitoba, Winnipeg, Manitoba Canada; 2 Wiyasiwewin Mikiwahp Native Law Centre, College of Law, University of Saskatchewan, Saskatoon, Canada

**Keywords:** Health, Resilience, Well-being, Meaning-making, Indigenous youth, Urban, Land, Nature, Canada

## Abstract

**Background:**

Population and environmental health research illustrate a positive relationship between access to greenspace or natural environments and peoples’ perceived health, mental health, resilience, and overall well-being. This relationship is also particularly strong among Canadian Indigenous populations and social determinants of health research where notions of land, health, and nature can involve broader spiritual and cultural meanings. Among Indigenous youth health and resilience scholarship, however, research tends to conceptualize land and nature as rural phenomena without any serious consideration on their impacts within urban cityscapes. This study contributes to current literature by exploring Indigenous youths’ meaning-making processes and engagements with land and nature in an urban Canadian context.

**Methods:**

Through photovoice and modified Grounded Theory methodology, this study explored urban Indigenous youth perspectives about health and resilience within an inner-city Canadian context. Over the course of one year, thirty-eight in-depth interviews were conducted with Indigenous (Plains Cree First Nations and Métis) youth along with photovoice arts-based and talking circle methodologies that occurred once per season. The research approach was also informed by *Etuaptmumk* or a “two-eyed seeing” framework where Indigenous and Western “ways of knowing” (worldviews) can work alongside one another.

**Results:**

Our strength-based analyses illustrated that engagement with and a connection to nature, either by way of being present in nature and viewing nature in their local urban context, was a central aspect of the young peoples’ photos and their stories about those photos. This article focuses on three of the main themes that emerged from the youth photos and follow-up interviews: (1) nature as a calming place; (2) building metaphors of resilience; and (3) providing a sense of hope. These local processes were shown to help youth cope with stress, anger, fear, and other general difficult situations they may encounter and navigate on a day-to-day basis.

**Conclusions:**

This study contributes to the literature exploring Indigenous youths’ meaning-making process and engagements with land and nature in an urban context, and highlights the need for public health and municipal agencies to consider developing more culturally safe and meaningful natural environments that can support the health, resilience, and well-being of Indigenous youth within inner-city contexts.

## Background

A growing body of evidence illustrates a positive relationship between access to green space or natural environments and peoples’ perceived health, mental health, resilience, and overall well-being [1–8]. Indeed, the assertion that nature can have beneficial or restorative health effects is well established; where reductions in stress, increased focus and concentration, improved general well-being, and heightened vitality are all positively related to the presence of and human connections with nature [9–12]. Research evidence also demonstrates that such connections with nature can result in decreased anxiety, aggression, and depression, while increasing a variety of measures of physical and mental health, optimism, self-esteem, vitality, and resilience or one’s ability to overcome stress and obstacles [13–17].

The concepts of nature or environment within this literature are often used interchangeably with a variety of related terms, such as the natural setting, greenspace, land, and natural environment [8]. Here nature is generally defined as an environment where some organic ecosystem processes are present, such as animals, trees, gardens, or vegetation in the landscape, plants, soils, water, rocks or air [13, 14]. Although definitions and outcomes of what constitutes health and well-being are complex and draw on different conceptual models, the World Health Organization’s (WHO) definition of health often informs research in this area as a state of physical, mental, and social well-being, not merely the absence of disease or infirmity [5, 6]. Similarly, the notion of resilience is often discussed as an aspect of coping, which implies the ability to ‘bounce’ back during adverse circumstances to support health outcomes [16–21].

Although the literature in this area is important and warrants serious attention, studies among general populations often advance narrow conceptions of nature, land, or environments as inanimate, secular spaces, often under the control, ownership, or care of human populations and municipal governments. To mobilize these approaches within Indigenous health contexts in Canada particularly, and global health more generally, offers limited conceptual and epistemological frameworks to understand the broader spiritual and cultural meanings attached to land and nature, and can obscure the historical processes of settler colonization, urbanization, and Indigenous knowledges influencing various conceptions of health, resilience, and well-being among Indigenous communities in Canada and globally [22, 23, 24].

### Indigenous perspectives of health, nature and resilience

Unlike most non-Indigenous peoples that might consider “land” or nature as something they own, a commodity to be traded, or an asset to make profit from, for many Indigenous populations perspectives of land and nature are much deeper [23–26]. In these contexts, maintaining connections with and having a relationship to nature and the “land” is not dependent on access to a literal material place or physical location, but can often involve symbolic or sacred representations, and spiritual relationships with broader more universalizing notions of “Mother Earth” [26–30]. In her study conducted with Anishinabek (Ojibway and Odawa) living in one First Nations community in northern Ontario, Canada, for example, Kathleen Wilson explored the ways in which land contributed to physical, mental, emotional and spiritual health, and noted that Indigenous peoples there often referred to land as “Mother Earth,” a provider of all things necessary to sustain life [31]. Land from this perspective, which harbours the trees, animals, and plants, is important in at least two respects. First, traditional lands are the ‘place’ of the nation and are inseparable from the people, their culture, and their spiritual identity [31]. Second, land and resources are the foundations upon which Indigenous peoples often intend to build and rebuild their economies and so advance self-determination, sovereignty, well-being, and the socio-economic circumstances of their peoples [29–31].

Historically, a relationship with nature, land or natural environment, has been a significant component of many Indigenous Peoples’ lives, identities, and cultures and is seen as an important determinant of Indigenous health [24–26, 32]. Health from many Indigenous perspectives is broader than merely physical health or the absence of disease, emphasizing the importance of well-being and a balance in four elements of life—the physical, emotional, mental, and spiritual—which are intricately woven together and interact to support health and well-being [23–25, 30, 33, 34]. Indigenous perspectives of health can also point to or are supported by strength-based processes of resilience that not only involve or draw on these four aspects of one’s being [35, 36], but also involve positive adaptation and resistance in the face of colonization, historical traumas, or structural violence, as well as current stresses, challenges, and demands [37, 38]. Indeed, research with diverse Indigenous Peoples attests that relationships with nature and land support all four elements of life, and this in turn can: enhance overall health, resilience, and well-being [28, 30, 31, 39]; improve self-esteem and self-efficacy [40], increase consumption of traditional foods [41]; foster intergenerational relationships [38]; reduce psychological distress [42]; and strengthen cultural identity and belonging [43]. For Indigenous youth in Canada’s north, as another example, beyond nature being a place where everything needed like animals to hunt to edible plants can be accessed, many also looked to nature as a place for spiritual connection, healing, and personal growth [26, 35, 40, 44].

Albeit diverse based on various unique histories, cultures, and languages, Indigenous perspectives of resilience are often grounded at a cultural level and are focused on the relationships that exist between community, identity, land, and culture across generations and geo-graphical settings [22, 45–48]. Indeed, notions of resilience here illustrate a strong link with culture and community and are largely based on processes of resistance to a history of oppressive colonial systems, discrimination, and loss. For example, in First Nations communities, resilience has been approached as a process or an ability of an individual to navigate toward resources that facilitate wellness and facilitate positive adaptation despite adversity [22, 36, 49–51]. For Indigenous youth within Canadian contexts, therefore, resilience is not just an individual’s capacity to cope with adversity, change or misfortune, but a community’s capacity to extend resources to sustain well-being and provide these resources in culturally relevant ways [33, 49–51]. In this way, young peoples’ views of resilience are often reflective of local culture and context in a holistic way, and “consist of a balance between the ability to cope with stress and adversity and the availability of community support” ([52], p. 5). Recent research has also shown that Indigenous youth perspectives of resilience draw on family and local environment as supporting factors that can be similar among cultures [45, 53].

### Current research objectives

Despite the research in this area, however, conceptual and methodological limitations persist. First, studies among general populations tend to involve limited definitions of health, resilience, nature, or greenspaces within urban environments. To mobilize these approaches within Indigenous health contexts can obscure the broader meanings attached to “land” and nature upheld by many Indigenous communities, including the historical and cultural perspectives of health, resilience, and well-being [22, 23, 25, 28, 30]. Second, previous research among Indigenous communities tends to restrict the conceptualizations of “land” or nature to rural home communities, such as reservations, northern and remote communities, or traditional and ancestral territories. When land has been subjected to health research in urban spaces explicitly, it typically employs narrow definitions that view land-based cultural practices as “out of place” or nearly “invisible” [45]. Less considerations, therefore, are given to how diverse groups of Indigenous Peoples’ maintain connections to land and nature within urban contexts to promote notions of health, resilience, and well-being they uphold [31, 43].

What we explore here is how health, resilience and well-being for Indigenous young people can be strengthened when connections to nature or land-based activity occur within urban contexts. We need to better understand the various ways that growing numbers of Indigenous youth living within cities understand their relationships with or access to natural environments, and how those relationships can support their local perceptions of health, resilience, and well-being. The research presented here critically engages these issues through a qualitative photovoice study conducted with urban Indigenous youth from Saskatoon, Saskatchewan in central Canada. Several “meaning-making” processes animated by context-person interactions and associated with youth perceptions of and day-to-day engagements with “land” and nature in their local urban contexts are explored [54, 55]. In so doing, we challenge common approaches to health research detailing connections with nature to consider Indigenous perspectives of “land,” health, and resilience, and at the same time examine some of the ways that contemporary Indigenous youth actively construct meaningful relationships with “land” and nature amidst their urban environments. In the end, we explore how connections to land and nature could inform public health interventions for contemporary Indigenous young people experiencing various social inequities within their urban environments.

## Methods

### Research framework

The research approach was informed by *Etuaptmumk*, a *Mi’kmaw* framework for “two-eyed seeing” where Indigenous and Western knowledge or “ways of knowing” (worldviews) are envisioned to work alongside one another [56]. The “two-eyed seeing” framework proposed by Mi’kmaw Elders Albert and Murdena Marshall was a means to bridge Western science and research with Indigenous knowledge and practice. Applied to our research team that was made up of both Indigenous and non-Indigenous researchers, this approach made space for open discussions regarding the crucial roles of both “ways of seeing,” improving our research rigour and overall understandings of urban Indigenous youth health, resilience, and well-being [57].

Situated within a social constructionist epistemological perspective, this research used a community-engaged qualitative approach bringing an Indigenous methodological research design together with a modified Grounded Theory (GT) methodology for data generation and analysis [58, 59]. An Indigenous methodology involves a paradigmatic approach where decisions concerning the choice of methods, how methods are employed, and how the data are analyzed and interpreted are shaped by Indigenous ontology and epistemology [59]. Indigenous ontology has been described as relational, placing value on not only knowledge itself, but on the relationships we share with it. Relationality is also central to an Indigenous epistemology, which characteristically sees researchers as connected to subjects of research and places value on subjectivity. Consequently, implementing this modified GT was necessary to create space for integrating aspects of an Indigenous methodology and “two-eyed seeing” that might otherwise conflict with the tenets of classic GT [58, 59].

The method of photovoice and photo elicitation were used to explore youths’ perspectives of ‘how’ and ‘why’ a connection to the land or nature in their urban contexts was important to them. Photovoice involves individuals taking photographic images to document and reflect on issues significant to them and how they view themselves and others [60–64]. We used photovoice because it is a collaborative, community engaged process that acknowledges the significance of giving voice to situated events and perspectives through photographs and a sense of “being there” [63, 64]. As an arts-based and Participatory Action Research (PAR) method, photovoice was a positive way of engaging young people and entering their worlds, fostered relationality between youth and the research team, and encouraged a storying of the sacred and creative aspects of one’s journey, as “the ceremony of art touches the deepest realms of the psyche and the sacred dimension of the artistic creative process” ([25], p. 46). By incorporating photovoice and understanding that youth need to be heard in a safe environment—which is not the case for many young people—we were able to promote knowledge generation that explored current and past assumptions about inner-city Indigenous youth and allowed them to ask creative or critical questions that may not have been uncovered or voiced otherwise [62, 63].

### Participants and sampling

A combination of purposeful and snowball sampling methods was used to recruit youth, self-identifying as Indigenous (i.e., Plains Cree and Métis), through partnerships with local youth organizations and a Community Advisory Research Committee (CARC) consisting of parents, Elders, and local youth who informed this research. The focus of sampling was placed less on generalizability and sample size, and more on sample adequacy so that depth and breadth of information was achieved as determined by thematic data saturation [58]. In total, 28 youth between the ages of 16 and 25 years (12 male and 16 female) who self-identified as being from Plains Cree (*n* = 21) and Métis (*n* = 7) cultural backgrounds participated. Written and verbal informed consent was obtained from all participants in this research. Consent was not obtained from the parents/guardians of minors (under the age of 18) who participated in this research. Youth 16 years and older were deemed able to consent to participate for themselves and this was approved by the University of Saskatchewan’s Behavioral Research Ethics Board (#14–141) and locally by the CARC.

### Photovoice procedures and interview protocols

This research was carried out within the inner-city neighborhoods of Saskatoon, Saskatchewan, at the Community Engagement Office, a satellite center of the University of Saskatchewan. Our project took place over an entire year and included four sessions of community-engaged photo taking data collection to explore changes in and perceptions of resilience and well-being over the seasons [57]. Based on consultations and engagements with our CARC, the intent was to capture diverse stories of challenge and resilience that could assist other youth in the community better navigate paths toward well-being.

At the start of each season, we engaged youth about their vision for the project, gave them digital cameras, and invited open talking circle discussions facilitated by our research team and Elders about the photovoice process. Youth were then invited to take pictures during a two-week period of different objects, people (with permission), or aspects of their lives that support their resilience, and general health, or well-being. Following each phase, talking circle interviews with the youth facilitated by the first, fourth, and fifth authors occurred to learn more about the meanings, interpretations, and experiences behind the youths’ photos. This approach is referred to as “photo elicitation” and purposefully focuses on the images as a point of conversation and dialogue, rather than centering on the youth themselves, in order to allow them to be more comfortable and open [59]. In following the guidance of our CARC and a “two-eyed seeing” approach, Cree and Métis Indigenous ways of life or “protocols” of smudging were offered before all gatherings or interviews and gifts of non-commercial tobacco to youth were followed in order to respect the sacred aspects and participatory nature of the research process [57, 59].

### Data analyses

In accordance with a modified constructivist GT approach [58], the core themes that emerged from the first interviews (*n* = 28) influenced the focus of subsequent interviews. Coding methods on the first round of interviews followed a constructivist GT approach [58] and were done by the first author. As codes, categories, and themes emerged, they were checked with the youth participants, CARC, and the research team (i.e, authors). This initial round of analysis informed a second round of interviews (*n* = 10) that was conducted by the research team to clarify the data and reach data saturation [58]. Ten youth were invited to participate in a second interview based on the strong themes of and strategies for resilience that emerged from their first interviews. Since we followed a “photo elicitation” method, individual youth photos were not coded [65, 66]. Following the two rounds of interviews, techniques and methods of constructivist GT were again utilized by the first author in subsequent stages of data analysis, including data reduction, data display, and conclusion drawing or verification [58]. Data summaries, coding, finding themes, and writing stories occurred as the data were being collected. Initial themes were coded using Dedoose software Version 8.1 (2018). Separate analytic files were constructed by the first author and checked for consistency by the research team, participating youth, and CARC. The data were examined repeatedly by all authors to capture and highlight the main themes and to create concise phrases that encapsulated the essence of the section, theme, or concept being expressed by youth [58, 59]. To enhance credibility and rigor, we employed peer debriefing among the research team where major themes were vetted by the CARC.

## Results

**Fig. 1:**
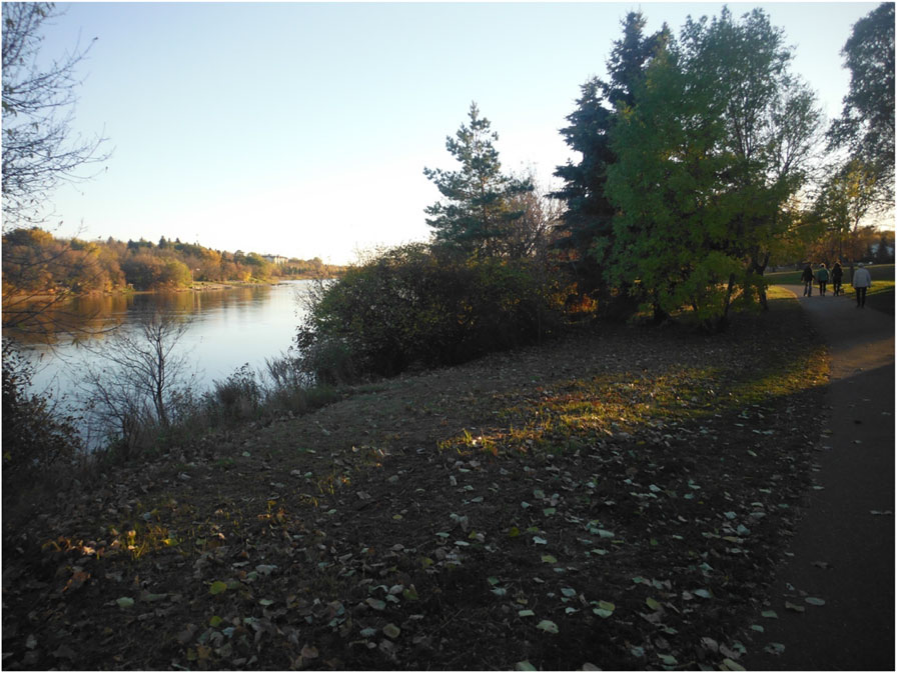
Youth’s photovoice image signified as “the river calms me down”

**Fig. 2:**
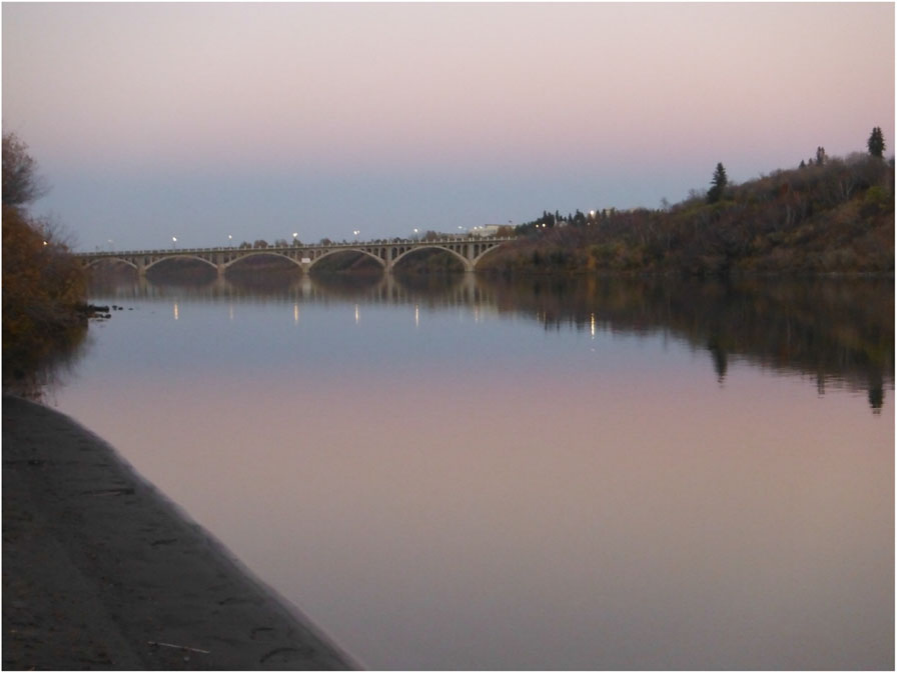
Youth’s photovoice image signified as “the river provides a sense of peace and positive memory”

**Fig. 3:**
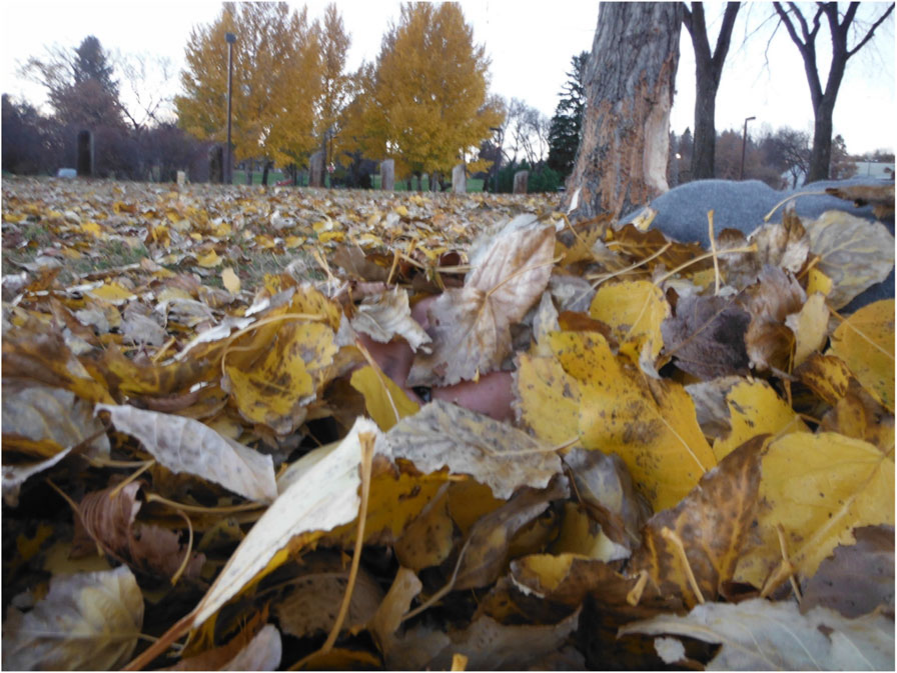
Youth’s photovoice image signified as “resting in the leaves and feeling at peace”

**Fig. 4:**
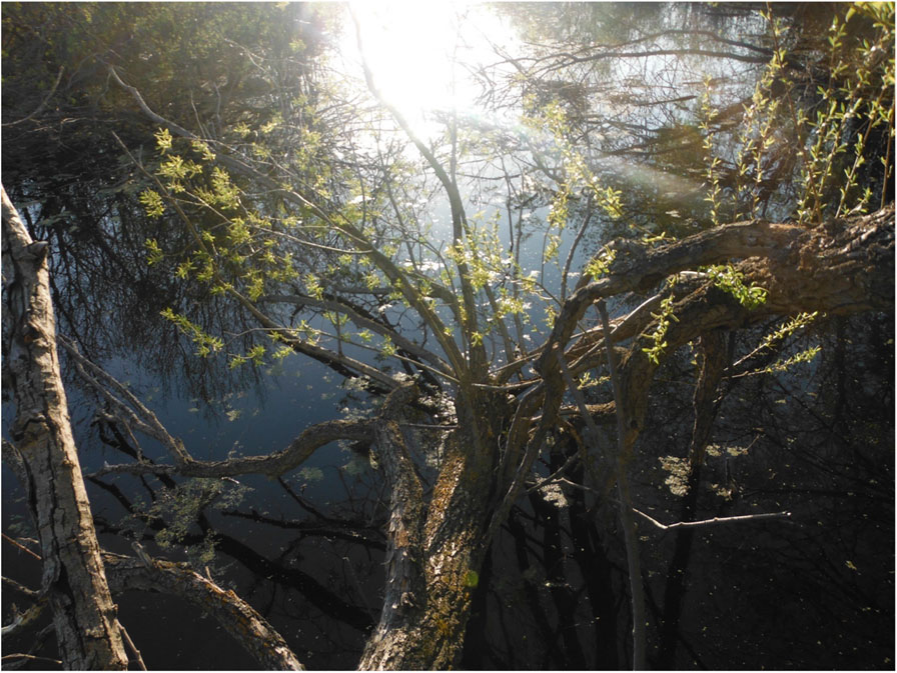
Youth’s photovoice image signified as “spring as a metaphor for change and growth”

**Fig. 5:**
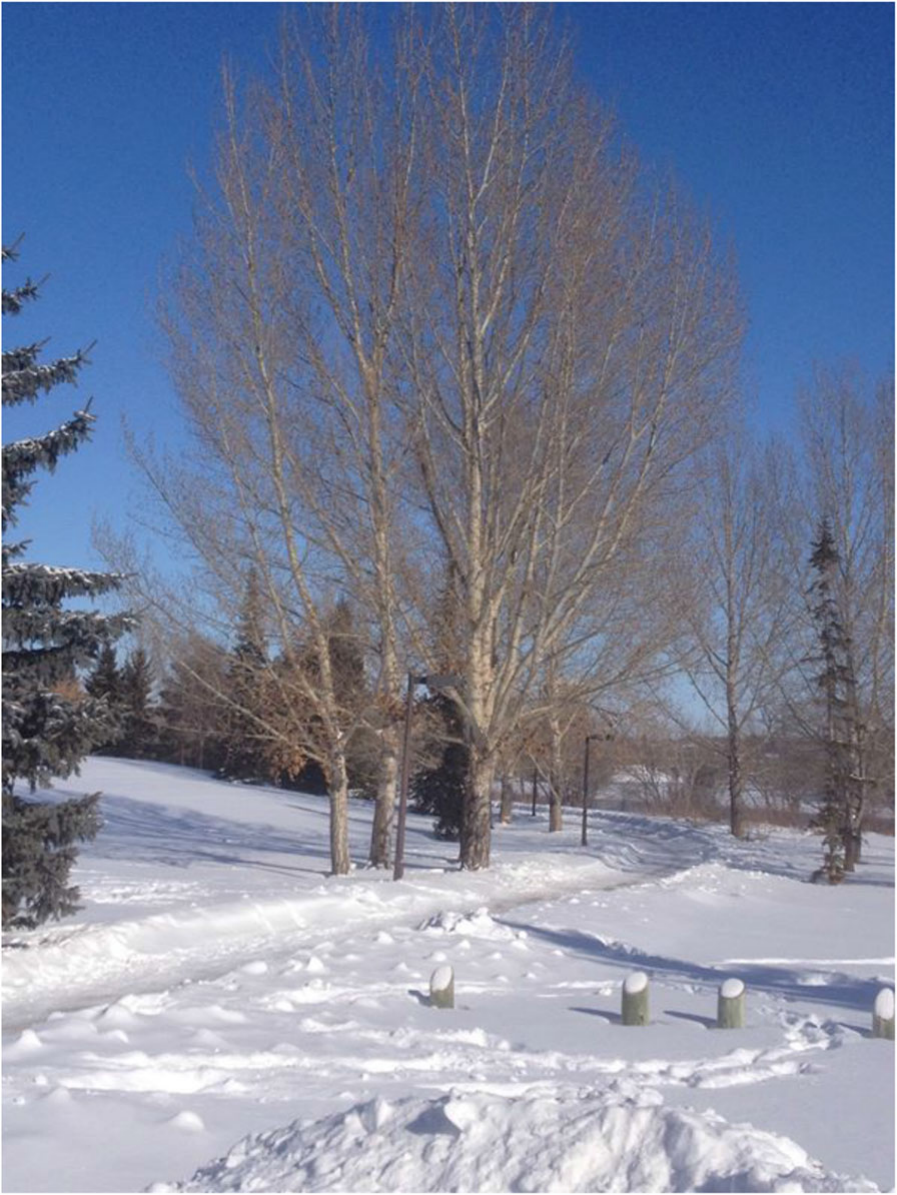
Youth’s photovoice image signified as “surviving winter”

**Fig. 6:**
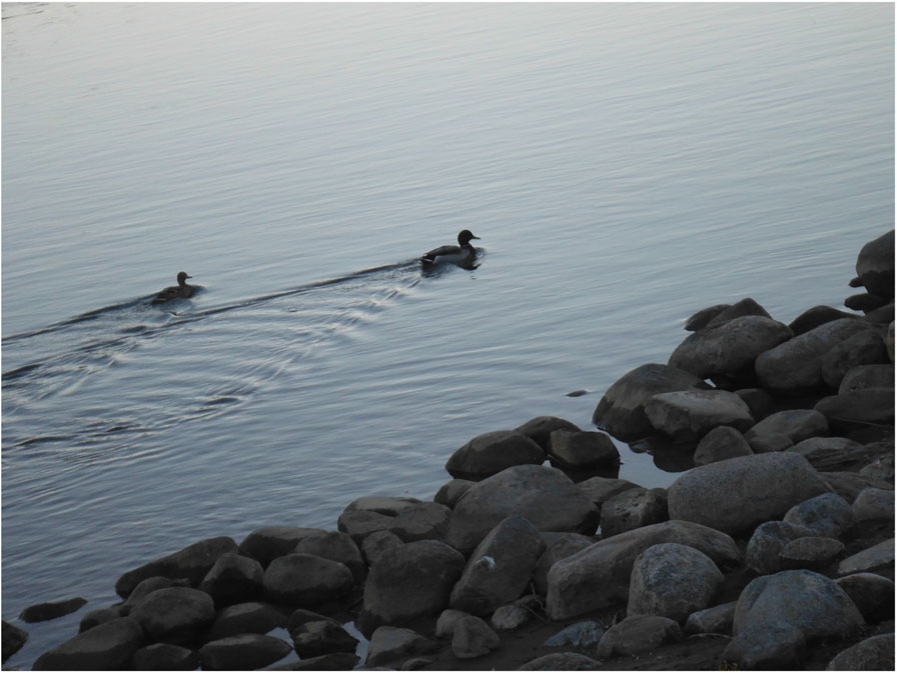
Youth’s photovoice image signified as “life lessons from nature’s animals”

**Fig. 7:**
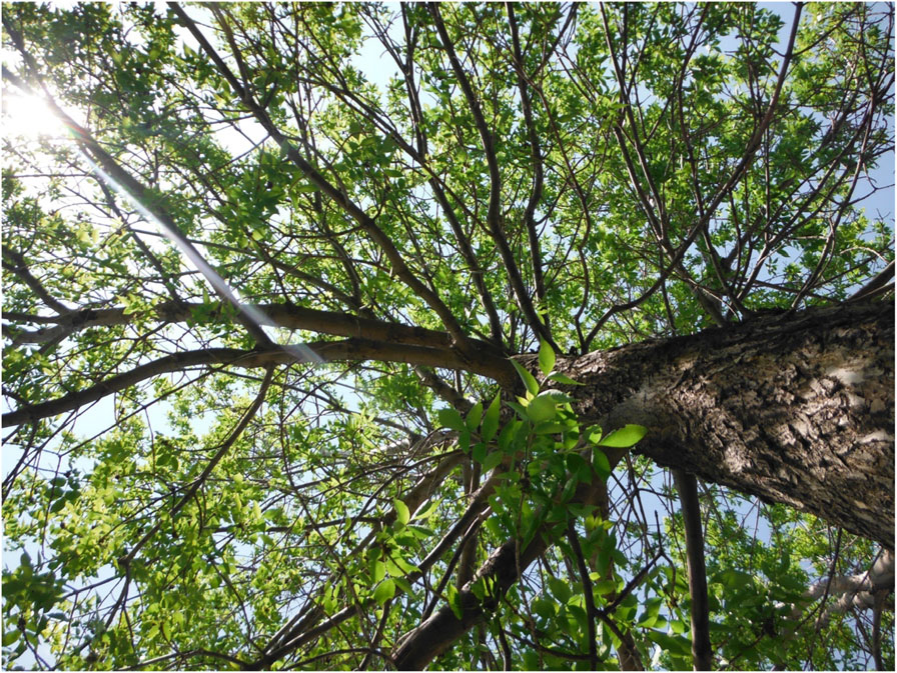
Youth’s photovoice image signified as “branching out and avoiding the bumps in life”

**Fig. 8:**
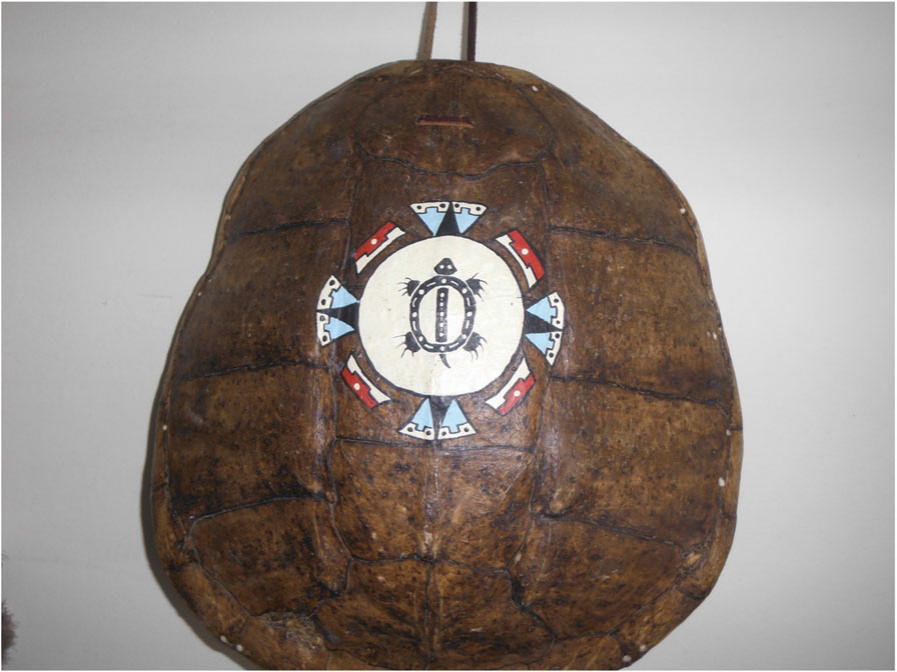
Youth’s photovoice image signified as “Turtle Island as our Mother Nature”

Analyses of youth stories and photos revealed complex processes of and navigations between contexts of distress and strategies of resilience and well-being. The photos included places and spaces in their urban environments (buildings, youth centers, schools, street corners), people (family, friends, partners), and ceremonial or cultural objects (traditional medicines such as sage and sweetgrass, drums, powwow dresses). Although the stories and photos that youth generated were diverse and covered a wide range of topics, a central theme that emerged in both the youth photos and stories were aspects of and relations with nature or the “land.” The findings explored here focus on “meaning-making” and context-person engagements with and connections to nature, either by way of being present in nature or by connecting with land in their local urban context, and how such connections fostered resilience and well-being in various ways [43, 54, 55, 62]. At the same time, we highlight how these connections helped young people cope with various stressors or difficult situations they may encounter and navigate on a day-to-day basis. Here we focus on three of the main themes and processes that emerged from the youth photos and follow-up talking-circle interviews: (1) nature as a calming place; (2) building metaphors of resilience; and (3) providing a sense of hope. Selected photos from the youth participants (Figs. 1, 2, 3, 4, 5, 6, 7 and 8) that correspond with and emphasize their thematic excerpts from the interviews are interspersed throughout.

### Nature as a calming place

For the youth in this research, nature generally involved aspects of their local environment unaffected by human intervention, design, or technology—the trees, plants, water, different seasons, animals, birds and other natural elements. Nature was primarily a positive force in the lives of these young people that was seen to drive away or protect them from negative experiences that they had to deal with at different points in their lives. When they are in the presence of nature or absorbed by natural environments within the city, the youth spoke about how it helped them to reduce stress, be distracted from pain or discomfort, and made them feel a deeper connection with their loved ones. In this way, the presence of nature brought about perceptual and emotional shifts towards a more calming and positive state of being. As one youth expressed, “When I’m upset, I think about stuff, like the water. To me, this kind of reconnects me. And at times, I let off steam by going for walks by the river…and here I feel happy, happy with Mother Nature.” As another youth similarly shared,

Whenever I’m like feeling really down or I just need to get away from everyone and just focus on myself, I go for long walks. And it’s really nice because I really appreciate nature and everything. So, it’s really calming.

Similarly, while many young people provided rich descriptions about the beauty and sight of nature, the sound of nature was also expressed as comforting. As another youth described,

When I am mad, it is nice when I just come down here by the river, ‘cuz like I said earlier, like it cools me down since my dad left. And sometimes I just take my shoes off and walk in the water, and then just listen to the water—splash in it sometimes like that’s what my dad and I used to do. So, it calms me down (Fig. 1).

To this young person, living without a father figure and not having a close family structure was the root of much anger, stress, and worries in life. As a kid, his dad used to take him by the river and play with him while going for long walks. Since his dad passed away, life has been harder for him. This youth expressed deep struggles at school, with other family members, and being teased and bullied by other youth. As a result, he regrettably at times takes out his frustrations on those around him, including family members, his girlfriend, teachers, or other students. Amid all these life struggles, connecting to nature and being by the river, in particular, was described as an important calming natural place that provided a sense of peace and positive memory. This sentiment was not unique, but was reflected in several youth narratives and photos. As another youth outlined,

I really like that I took pictures by downtown and by the river landing, because that’s where I like to go when I’m stressed out or just like my alone time. I just really like it. The sky itself is really blue and it gets like lighter. It’s really beautiful and calming (Fig. 2).

These experiences and narratives expressed by the young people we worked with reveal deep connections to nature—represented often by the land and river—and thereby illustrate how positive, meaningful relationships between their interactions with natural environments and their well-being can occur within urban contexts. The feelings of calming serenity which nature offers cannot be derived by attending support programs alone, socializing with friends or family, or even participating in cultural activities and school initiatives. Rather, it is the person-context interactions of young people with nature and the "land" in their urban contexts that animated meaning-making processes and emotional shifts towards more positive and calming affective experiences.

### Building metaphors of resilience

For the youth in this study, nature is also seen as a spiritual force that can guide people to do or not do certain actions in life. In most cases, the youth described such images, stories, or teachings coming to them from nature as a lived and embodied metaphor [55]. This notion is also expressed by Gregory Cajete as a “nature or metaphoric mind,” wherein he suggested that “as the rational mind develops, the metaphoric mind slowly recedes into the subconscious” where it lies dormant until “its skills are called upon by the creative play and imaginative reverie, or in dreams and stories” ([25], p. 28). Interactions with and connections to nature here thus become one such meaning-making process to awaken the imaginative stories and creative interplay supporting youth well-being within their inner-city environments. Metaphor thus involves a process of discovery or invention, and is essentially a creative meaning-making process that can become a tool for working with challenging experiences [55]. In this way, metaphors can also be thought of as being embodied insofar as they provide idiosyncratic ways of acting on representations and of making presentations to others that link individual acts with collective sense-making [54].

Reflecting this notion of embodied metaphor, one youth for example felt that the season of fall—with the brightly colour leaves falling from the trees—represented new beginnings and an opportunity to start over or “re-do” previous hardships or negative life experiences. This metaphor, at times, offered peace to her mind, made her feel rested, and gave her an inner sense of strength, despite unexpressed anger from a disturbing childhood. As she shared,

In this photo, I wanted to show how the leaves have fallen and now they are resting, and I was just sitting there and resting with them and feeling at peace. Because I am settled here, kind of like how the leaves are settled. I feel more connected to myself. I don’t feel lost and angry, like how I did as a kid. Seeing this in nature helped me see this in myself (Fig. 3).

Meaningful person-context engagements with nature and the different transformations of the seasons of the year were often seen and expressed as powerful metaphors and guides informing particular cultural practices and ways of action. When the time of the winter solstice appears (often a time of reflection in Cree culture), for example, young people often interchangeably felt more “closed,” “dark,” and “cold,” as the snow covers their environment. Yet, no matter how dark and cold the winter may be, the youth outlined and recalled how “there will always be a springtime to follow.” Drawing on this imagery, the youth often connected how in life there may be high points and low points, but Natural Law continues to advance and “things will work out,” that “life continues to move forward,” and “changes and grows.” The spring, then, can often embody and reflect metaphorically the flux of life as experienced by youth, and how to prepare or act with hope to the future amidst such aspects of change and transformation. In this spring season (often known as the renewal time of year), the leaves are growing back on the trees and there is more light and hours in the day. In this way, spring can be a reminder that any darkness in one’s life is always temporary and to be ready and adjust in such a way to explore and maximise the long hours of the day in order to accomplish goal. As another youth expressed,

In the spring, when you see the leaves budding, it’s the preparation for change. Preparation for change...it just means new growth, new opportunity. So, it’s like the beginning of these goals that we intend to do through this stretch of time where the sun is closest to us (Fig. 4).

Again, for this young person, seeing the trees change with the onset of each season shows that the world around him is regenerating and resilient. Natural Law and spiritual interactions with nature then teaches him to be resilient and to know that change and renewal are a natural cyclical aspect of life. These embodied metaphors of seasonal change have helped this youth adapt to changes and “go with the flow” of the seasons. As another youth similarly elaborated,

Seeing the trees change shows change in life because it identifies the seasons … A tree changes or dies out in the winter or these cycles that make life evident, and shows us that regrowth is possible because it comes back every year … Just that comfort in knowing that the nature around me is resilient, so I must be resilient.

Herein, just like an Elder that guides and gives youth spiritual teachings and lessons in life, relationships with nature, represented by the seasons, is also acting as a teacher for these young people. The teachings learned through person-context contact with and meaning-making observations of nature within an urban landscape are seen as important for these young people—as they can guide and support youth that face and cope with the daily struggles of inner-city life.

As mentioned, although youth engagements with land and nature support aspects of resilience and emotional well-being, there is also a complexity in such human-nature relations that can have negative influences as well. Given the cyclicality of seasons and the changing patterns of vulnerability or adversity reflected therein, many young people described the physical burden of colder Canadian prairie seasons on the body as well as the emotional burden of family grief in the winter. “Winter is a hard time for me” as one youth shared, “it’s a time where everything slows down, and we can become isolated and lonely sometimes” (Fig. 5). Or as another expressed, “The cold outside sometimes makes the people cold inside, less willing to stop and say hi, you know.” Despite these expressions, however, youth again described a capacity to act when referring to how colder seasons prompted a shift into an embodied “survival mode.” In this way, youth displayed processes of “surviving” or resilience that can protect or support them from the physical, emotional, and metaphorical burdens or experiences of winter, while also providing imagery of impermanence inherent within life’s challenges.

### Providing a sense of hope

Building on these ideas of an embodied metaphor and meaning-making processes, another theme of nature-person interactions that emerged from the youth photos and stories involved providing a sense of hope. The notion of “hope” here was primarily about a future time orientation where youth looked beyond current challenges to hoped-for positive opportunities [18]. For one youth in particular, nature in the form of a bird reminds her that life is not a straight road, but that obstacles and challenges will always exist, but can be overcome. As she illustrated,

But when I see these ducks being in relationship, I’m all like oh, they are living life, being all cute. I think ducks mate for life, and they are just happy little ducks here in this photo They have like this positive little bond of being cute little ducks swimming along, just living life (Fig. 6).

For this youth, she had been in previous unhealthy relationships, which constitutes the primary reason for her ups and downs in life. During her high school days, she got into her first relationship at 17 years old. As she explained, her boyfriend controlled her life. She was abused mentally, emotionally, and eventually it got to the extent of sexual abuse. It was a negative relationship which made her feel sad and upset about life, a story not uncommon among the youth in this research. As a result, she lost concentration in school and was completely consumed with her own issues and negativity. Feeling embarrassed and ashamed, she kept these situations to herself, and never spoke about these challenges with friends in school or family members. This was one of her lowest times in life. Despite facing these challenges, however, the thought and interactions with ducks and animals living in nature, gave her a sense of hope to believe that healthy relationships can exist. To this youth, nature became a model or teacher for healthy relationships, which brought an important sense of hope and positivity into her life.

In a similar way, other youth described a sense of hope through the imagery of a tree. Just as the tree grows by branching out and expanding, these youth expressed that they should not give up in the face of difficulty but let their experience “branch out” to avoid hitting a “bump” in the road of life. As one youth explained*,*

The tree grows with you. Like, as you’re going along with life you may hit a bump in the road, and you’ll go one way as a result. And then when you come back, like you’ll keep making branches along the way. And since that’s your life, the trees need all those branches to live, so then they go all the way up … So, you need all the bad stuff in your life to just be able to learn and go through … it’s never like the end because you still have a lot more growing personally (Fig. 7).

For this young person and others like him, he had experienced tough phases in life which he termed as “blockages” that prevented him from seeing the future and living the life he wanted. As a young teenager, his one-month old cousin had a stroke. During this entire period of ill-health, the cousin was the focus of everyone in the family. Along with his mom, they would always visit him both at home and at the hospital making sure and providing everything he needed. He was equally the babysitter that would take care of all the other children around the home. Constantly thinking about the cousin’s situation, whether he was alive or if the night before was going to be last time he was ever going to see him, he lost focus on school and on other aspects of life. He felt extremely upset at the situation and eventually stopped building friendships or even hanging around friends. About a year after the cousin’s birth, he ultimately passed on. This was the hardest time, indeed, truly a rough and hectic period for him. As much as it was difficult, this experience was seen and interpreted as an important life lesson and meaning-making process. Having the knowledge and imagery of the tree in mind and how it grows through “bumps” and “struggles” was really important for this youth, as it made him more at peace with the negative experience, feeling less angry, and allowed him to focus on his future. Even though he went through a rough path, he chose not to give up, but instead “branched out” and, like the different branches of the trees, moved out and grew in different directions.

Along these same lines, another young person illustrated how cultural teachings of hope for the future reinforce human-nature embodied relations through a sacred turtle shell gifted to him. The turtle shell also connected this youth to processes of gift giving, traditional dancing, expressing cultural pride, and learning more about the “land”:

This is my shield for my outfit – this turtle shell. It was given to me as a gift because I didn’t have a shield and a lady recognized it. I use it a lot. It’s a very nice gift. I like dancing [powwow] with it. I get a lot of good comments about it. A lot of people admire the turtle because, Turtle Island represents our mother, you know like Mother Nature, and I’m just going to take care of it and keep it close because it was given to me for a reason (Fig. 8).

Through these teachings, the young person’s relationship with nature in general, and the sacred turtle shell in particular, provides “reason” or purpose to warrant renewal, growth, thriving, and resilience. This young person demonstrated how spiritual connections to nature and “land” become embodied through cultural teachings that extend place to include “Turtle Island,” which encompasses not only Saskatoon but the entire North American continent. This extension shows how youth can make or create space for culturally rooted stories, teachings, and conceptions of land and nature to function materially and spiritually within urban cityscapes, and thus becomes an important strategy of identity re-creation and hope for the future [28].

## Discussion

This study contributes to the literature exploring Indigenous youths’ meaning-making process and engagements with land and nature in an urban Canadian context. Through their photos and stories about those photos, youth in this research revealed how local encounters with nature were constructed through day-to-day affective activities that changed and were fluid over time, leading us to interpret these processes through everyday acts of “meaning-making” [40, 43, 54, 55]. A move to analyze “meaning making” and youth engagements with nature in urban contexts not only considers temporal orientations, but also emphasises conceptions of situated instances of “ecological resilience” through multiple, overlapping, and contradictory processes of renewal and growth [18–20, 54]. Nature was seen here as endowed with sacred or spiritual meanings and embedded in relations that served as powerful embodied metaphors that helped youth to mitigate distressing life experiences. The trees, plants, and animals which inhabit the “land” were also highly personal beings which formed part of young people’s social and spiritual universe, and taught youth important life lessons [43, 44]. The youth also learned through the seasons that change is a natural part of life, and therefore, in the face of change and difficulties, they have to adapt and move on to accomplish a goal. Overall, then, this exploration builds on and can inform strengths-based work in the area of urban Indigenous youth well-being and resilience [67, 68].

### Resilience, “land,” and meaning-making

The initial focus of resilience on the qualities of an individual has evolved in the last two decades [19, 20, 21, 51]. Indeed, previous literature exploring resilience among youth has often conceptualized resilience as a static outcome associated with risk and protective factors, and not typically as a dynamic and contextual process in dialogue with local worlds and environments [19, 37, 51]. From Indigenous perspectives, Burack and colleagues have criticized what they call “simplistic linear risk models of a specific predictor to a specific outcome” as inadequate for understanding real-life complexities for Indigenous youth ([69] p. S18). Population health and resilience research has also been criticized for theoretically placing the onus on Indigenous or other marginalized youth to become resilient or healthy while not explicitly interrogating historical and contemporary forms of structural disadvantage (i.e., colonization) that permit inequity, marginalization, and exclusion [22, 35, 44]. In these contexts, therefore, it remains crucial to understand youths’ resilience from a relational worldview that encompasses the context, the mental, emotional, physical and spiritual connections with land and nature, as well as the unique interactions and structural impediments to well-being and resilience [35].

Taking these issues into consideration, research now illustrate that much of what seems to promote resilience amidst adversity originates outside of the individual—in the family, the community, the society, the culture, and the environment [18–22, 35, 44]. As a result, population and community-based health researchers have explored resilience promoting factors beyond the individual level, to consider from a social ecological perspective these different levels and youth-environment interactions [22, 46–49, 51, 70]. Similarly, Indigenous-specific resilience research have also explored resilience-building process that emphasized the importance of culture, identity, community, family, spirituality, and Indigenous worldviews as integral factors that enhances resilience [18, 48, 67, 68]. Although we do not deny the individual role of these resilience factors in facilitating better health and wellbeing of youth [51], it remains important to not only understand the intersecting relationships among these individual, structural, and social factors involved in resilience, but also to examine the relatively unexplored areas of Indigenous youth meaning-making connections to “land” or nature within urban contexts and how they can also play a role in supporting resilience and well-being.

### Decolonizing urban environments

Due to long histories of settler colonialism, dispossession, oppression, and contemporary forms of racism, certain aspects of urban environments, like city parks or walking trails, are not always safe spaces for Indigenous young people. In such ways, young people continue to face subtle and overt forms of discrimination, racism, or do not enjoy basic rights that other non-Indigenous youth populations living within urban environments may take for granted or are privileged to assume are normalized [29, 32, 71, 72]. Indeed, many young people in our research expressed how, in their 18 or so years of life, they still have not crossed the bridges across Saskatoon’s river from the west to the east sides of the city, and do not feel like they belong in certain parks or green spaces that are primarily associated with the dominant “White” population of the city. Clearly, then, not all nature spaces are equal. Such disparities in the built environment of a city not only have direct impacts on the social determinants of health and the individual development of young people—including economic capacity or opportunity and psychosocial domains of belonging and safety—but can also affect young peoples’ ability to be resilient and surmount life struggles [12, 61, 64]. When youth are able to navigate within and engage with nature in culturally relevant and meaningful ways, the natural environment can provide an empowering space, offer a sense of peace and hope, positive memories, and foster an inner strength to forge ahead amidst life’s challenges and obstacles [30–33, 39, 43, 62].

Several authors have argued that Indigenous connections with land and nature can represent “decolonizing” initiatives, drawing on a spiritual identity and cultural history to challenge the spatial arrangements of settler colonial legacies [28–31]. What we observe in this research is how youth are constructing and building such connections within their local urban spaces and thereby challenging assumptions of Indigenous space, identity, and notions of belonging that are only rooted in ‘ancestral lands’ or rural reserve communities “out there” beyond the city limits [43]. Indeed, current “land back” movements, local forms of organizing, protest, and discourse burgeoning across urban Canadian centers over the last several years can been seen as examples of such strategic decolonizing actions that assert self-determination and enact resilience by occupying collective space that was not historically “safe” or culturally represented as “Indigenous.” This research adds to these perspectives by illustrating how contemporary Indigenous young people within inner-city contexts are building connections with nature or “land-making” that can, in various ways, further support their resilience and well-being [43]. In this way, the local modes of meaning-making and connections with the land we highlight here can be seen as a part of the broader national Indigenous rights activism, political resistance, expressions of agency and sovereignty, presence, and self-determination regarding protection of the sacred “lands” and the importance of land claims at national and global levels [32]. Indigenous self-determination and land rights in Canada and globally, both at the macro levels of settler state politics and the micro levels of the day-to-day lives of inner-city youth, will only continue to be of relevance as increasing numbers of Indigenous peoples continue to migrate to and find a “home” within urban centers [72].

Given these findings, provisions for and access to natural spaces, “land” or nature-based approaches to population or community health are not only important generally [3], but can inform holistic health approaches to supporting the health, resilience, and well-being of Indigenous youth within urban settings. This is also important given that, due to the histories of colonization, ongoing industrialization, and increasing urbanization, several Indigenous young people within urban contexts can have limited exposure to or culturally meaningful ways to engage with nature and the “land” [51, 52]. Looking forward, in urban settings and culturally “safe” development, then, these natural spaces can be better offered through accessible and decolonizing city parks, community gardens, and youth cultural spaces that are co-designed with the young people that are intended to access them [29, 61, 71, 72]. Future research could also explore further what decolonizing processes in the local space could mean or look like as a form of population level structural intervention in order to ensure young people within the city are included, belong, feel welcome, and have opportunities to construct meaning and enact resilience during positive interactions with nature and the “land” [31–33, 39, 61, 71, 72].

### Photovoice and “two-eyed seeing”

The use of photovoice method combined with *Etuaptmumk* or a “two-eyed seeing” approach in this study remains important as it allowed youth to actively engage in the research process and connect visual images with their lived experiences or social realities [56, 57, 62–64]. Youth were also able to share personal knowledge about particular issues that were difficult to express with words alone [65, 66]. Additionally, photovoice methods presented opportunities for youth to reflect upon their interactions with nature and ways through which such interactions contribute to their resilience, overall health and well-being. Reflecting back to the “nature or metaphoric mind” that Cajete outlines, photovoice as an arts-based methodology can also become a special “skill” or activity that invites the creative play or imaginative reverie and allows the metaphoric mind to flourish [25]. By uncovering and amplifying Indigenous youth perspectives about connections to nature and its contribution to their general well-being and resilience through photography, this method not only served as an empowering approach for youth, but also worked as a powerful tool to visualize Indigenous concerns and facilitate discussions on needs assessments of Indigenous youth in urban settings [64, 71]. This research and a “two-eyed seeing” methodological approach can inform appropriate population health interventions or action to improve the resilience, health and well-being of urban Indigenous youth in the years ahead.

## Conclusion

Indigenous youth connections with land and nature may be a hidden strength or source of resilience and well-being that has not been adequately explored or understood within urban contexts in previous population or public health literature. Perhaps due to assumptions that “land” and nature for Indigenous young people are to be accessed “out there” in rural contexts and not “here” in the city, many of the positive health associations and meaning-making processes youth build with nature in urban places may be lost [43]. Given the positive impact and benefits which connections with nature can offer many youth in urban settings, public health and municipal agencies should ensure the natural environments are conserved, expanded, and made more culturally safe and meaningful so that Indigenous youth can easily connect to nature which guides and allows them to construct meaning in their lives. By ensuring that the quality and health of our natural environment is upheld, and parks and natural spaces are widely accessible, nature could therefore serve to be a key component of the urban health infrastructure that has the capacity to support resilience, the broader social and environmental determinants of health, and address the various health needs of Indigenous youths, other subgroups, and the broader population as a whole [1–7]. With this in mind, however, it is important to maintain an appreciation for deeper meaning and significance with which “land” and nature are appreciated within many Indigenous cultural worldviews [22–25]. Hence, caution should be taken when engaging cross-cultural collaborations during public health research and interventions to manage these paradigmatic or cultural differences sensitively and respectfully.

## Data Availability

The datasets generated and/or analysed during the current study are not publicly available because of privacy concerns; participants are potentially identifiable due to the small sample and overall population size, and because of the qualitative nature of much of the data. The datasets are potentially available from the corresponding author on reasonable request.

## Abbreviations

CARC: Community Advisory Research Committee
GT: Grounded Theory
